# Immunoinformatics design of multi-epitope vaccine using OmpA, OmpD and enterotoxin against non-typhoidal salmonellosis

**DOI:** 10.1186/s12859-023-05183-6

**Published:** 2023-02-24

**Authors:** Babak Beikzadeh

**Affiliations:** Department of Cell and Molecular Biology & Microbiology, Faculty of Biological Science and Technology, University of Isfahan, Isfahan, Iran

**Keywords:** Non-typhoidal *Salmonella*, OmpA, OmpD, Enterotoxin, Immunoinformatics, Multi-epitope vaccine

## Abstract

**Background:**

Non-typhoidal *Salmonella* (NTS) is one of the important bacteria that cause foodborne diseases and invasive infections in children and elderly people. Since NTS infection is difficult to control due to the emergence of antibiotic-resistant species and its adverse effect on immune response, the development of a vaccine against NTS would be necessary. This study aimed to develop a multi-epitope vaccine against the most prevalent serovars of NTS (*Salmonella* Typhimurium, *Salmonella* Enteritidis) using an immunoinformatics approach and targeting OmpA, OmpD, and enterotoxin (Stn).

**Results:**

Initially, the B cell and T cell epitopes were predicted. Then, epitopes and suitable adjuvant were assembled by molecular linkers to construct a multi-epitope vaccine. The computational tools predicted the tertiary structure, refined the tertiary structure and validated the final vaccine construct. The effectiveness of the vaccine was evaluated via molecular docking, molecular dynamics simulation, and in silico immune simulation. The vaccine model had good binding affinity and stability with MHC-I, MHC-II, and toll-like receptors (TLR-1, 2, 4) as well as activation of T cells, IgM, IgG, IFN-$\gamma$ and IL-2 responses. Furthermore, after codon optimization of the vaccine sequence, this sequence was cloned in *E. coli* plasmid vector pET-30a (+) within restriction sites of HindIII and BamHI.

**Conclusions:**

This study, for the first time, introduced a multi-epitope vaccine based on OmpA, OmpD and enterotoxin (Stn) of NTS that could stimulate T and B cell immune responses and produced in the prokaryotic system. This vaccine was validated in-silico phase which is an essential study to reduce challenges before in vitro and in vivo studies.

**Graphical abstract:**

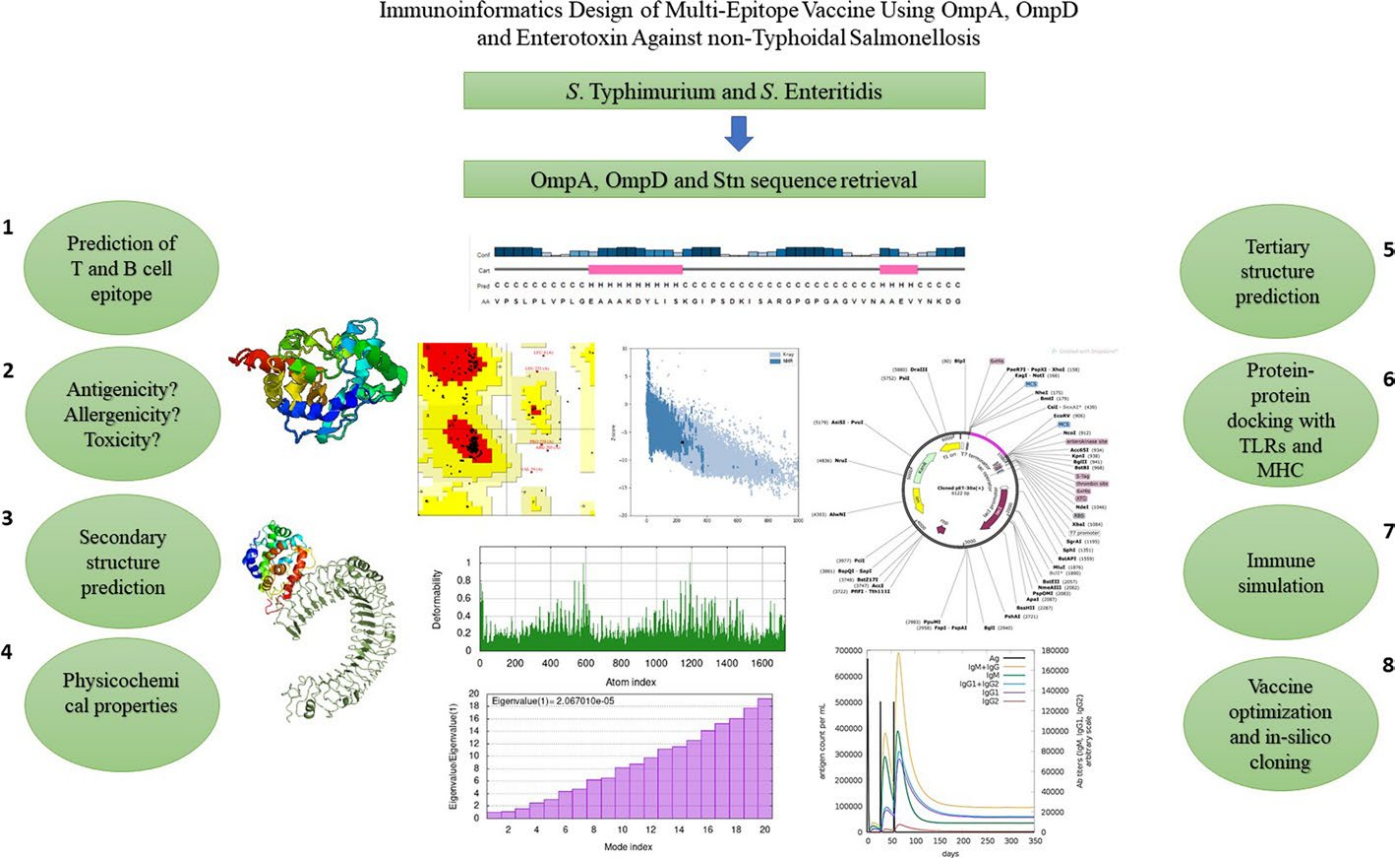

## Introduction

Non-typhoidal Salmonella (NTS) is a major cause of bacterial enteritis with 93.8 million diseases and 155,000 deaths each year [1]. In European Union (2020) Salmonellosis was the second most reported zoonotic disease with over 91,000 cases and an economic burden of €3 billion annually (https://www.efsa.europa.eu/en/topics/topic/salmonella). According to the CDC report, about 40,000 cases are registered annually in the United States (http://www.Cdc.Gov/salmonella/wellknown/index.Html). Two major *Salmonella* serovar related to the foodborne disease are *Salmonella enterica,* serovar *enteriditis* and *Salmonella enterica,* serovar *typhimurium* which are known as NTS [2]. Salmonellosis symptoms are often self-limited, and most patients recover without the need for special treatment. However, the resulting dehydration can occasionally become severe and life-threatening, particularly in children and elderly people [3, 4]. Studies have highlighted that in addition to diarrheal: fever, watery stool, stomach cramps, meningitis, and sepsis were observed in patients [5]. Regardless of the complicated processes involved in diarrhea induction, *Salmonella's* enterotoxin (Stn) is responsible for diarrhea [6, 7]. Moreover, controlling *Salmonella* infection is difficult due to the bacteria’s tolerance to environmental stress, its multi-drug resistance and wide distribution [2]. Antibiotics are the main choice for NTS therapy although they are no longer advised due to the increased risk of antibiotic resistance and adverse effects [5]. On the other hand, NTS suppresses humoral immunity, particularly antibody response and causes chronic infection in patients [8]. Furthermore, *Salmonella* invades macrophages and escapes from phagocytosis. *Salmonella* reduces germinal center formation and survival of IgG secreting-plasma cells. Since NTS infection remains a severe health problem, vaccine development is essential to avoid loss of immune responses [8].

Recently, several vaccines candidate has been developed for NTS including live-attenuated, subunit-based, and recombinant antigen-based vaccines [9]. The WT05 was the live attenuated vaccine that was tested in humans but did not have a good chance for further usage [10]. The outer membrane vesicles (OMVs) containing O-antigen are another vaccine candidate in the pre-clinical phase [11]. According to WHO report, the development of bivalent vaccine would be necessary to protect people against the predominant serovars *S*. Typhimurium and *S*. Enteritidis (https://www.who.int/teams/immunization-vaccines-and-biologicals/diseases/nontyphoidal-salmonella-disease). Furthermore, researchers discovered that the outer membrane proteins (Omp) include a class of proteins known as porins, which induce antibody response and cell-mediated immunity [12]. The major porins of NTS are OmpC, OmpF,

OmpD and OmpA. Evidence indicated that OmpD and OmpA are the highly conserved and abundant porins in *S*. Typhimurium and *S*. Enteritidis [13–15]. Since the vaccine production process faces several challenges, including time and considerable financial resources and also the efficacy of vaccines based on classical methods (killed or attenuated vaccines) must be evaluated on a specific host. Nowadays, the combination of proteome and immunoinformatics is employed to assess and increase vaccine efficacy with low costs. Although several in silico vaccine investigations were performed on NTS whole genome and Omps (OmpC, OmpF,

OmpD), but no commercial vaccine is available so far [5, 9, 16]. In these cases, vaccine design strategies focusing on the whole genome or proteins led to increased antigenicity and a higher risk of allergies [5]. Therefore, for the first time, the epitope sequences of the two most prevalent Omps (OmpA and OmpD) from *S*. Typhimurium and *S*. Enteritidis, as well as their enterotoxin (Stn), were investigated in order to construct bivalent multi-epitope vaccine.

## Results

### Prediction of T lymphocytes epitopes

First, the OmpA, OmpD and Stn were analyzed to identify their amino acid sequence similarity between *S*. Typhimurium and *S*. Enteritidis. Results of this analysis show more than 99% similarity in OmpA, OmpD of *S*. Typhimurium and *S*. Enteritidis while no considerable similarity was seen for Stn between these bacteria.

According to IEDB server results, in total 4177 epitopes for CTLs and14413 epitopes for HTL were predicted. The best epitopes were chosen for further analysis based on their high binding affinity, antigenicity score, non-allergenicity, non-toxicity and immunogenicity as illustrated in Tables 1 and 2.

**Table 1:** Predicted CTL epitopes from OmpA, OmpD and Stn proteins to design multi-epitope vaccine construct with their immunogenic properties

Protein	Allele	Start	End	Epitope	IC50	Antigenicity Score	Immunogenicity score	Allergenicity	Toxicity
OmpA	HLA-C*03:03	4	12	TAIAIAVAL	1.029461	0.6639	0.3067	Non-allergen	Non-toxic
OmpD	HLA-C*03:03	263	271	AAGDAFIAN	9.367204	0.5603	0.3168	Non-allergen	Non-toxic
Stn *S*. Typhimurium	HLA-C*03:03	76	84	RLIRREPQL	14.50808	0.5306	0.16289	Non-allergen	Non-toxic
Stn *S*. Entritidis	HLA-C*03:03	149	157	EAIFTPYFT	8.252965	0.8941	0.25364	Non-allergen	Non-toxic

**Table 2:** Predicted HTL epitopes from OmpA, OmpD and Stn proteins to design multi-epitope vaccine construct with their immunogenic properties

Protein	Allele	Start	End	Prediction sequence	IC50	Antigenicity Score	Allergenicity	Toxicity
OmpA	HLA-DRB1*04:05	241	255	ALDQLYSQLSNLDPK	39	0.7209	Non-allergen	Non-toxic
OmpD	HLA-DRB5*01:01	287	301	GLRPSIAYLKSKGKN	0.08	0.7573	Non-allergen	Non-toxic
Stn *S*. Typhimurium	HLA-DRB1*01:01	36	50	PTFFCLYANRSLAAN	10	0.7020	Non-allergen	Non-toxic
Stn *S*. Entritidis	HLA-DRB1*07:01	78	92	EIQLRFTANETLKRI	26	1.0701	Non-allergen	Non-toxic

### Prediction of linear B cell epitopes

The linear B cell epitopes were predicted using ABCpred server with (16 mer) length and cut-of binding score > 0.51. With these criteria, epitopes with higher scores from OmpA, OmpD and Stn were chosen for vaccine construction, which is listed in Table 3.

**Table 3:** Predicted B cell epitopes from OmpA, OmpD and Stn proteins to design multi-epitope vaccine construct with their immunogenic properties

Protein	Epitope sequence	Start position	Score	Antigenicity score	Allergenicity	Toxicity
OmpA	DYLISKGIPSDKISAR	287	0.89	0.8497	Non-allergen	Non-toxic
OmpD	AGVVNAAEVYNKDGNK	16	0.96	1.0182	Non-allergen	Non-toxic
Stn *S*. Typhimurium	ANRSLAANRMNSVQVQ	43	0.84	0.7743	Non-allergen	Non-toxic
Stn *S*. Entritidis	SGKGIAPDLVEAIFTP	139	0.93	0.6088	Non-allergen	Non-toxic

### Population coverage analysis of the selected epitopes

Global population coverage analysis for 8 selected epitopes (4 CTL and 4 HTL) shows 100% and 95.85% of people respond to CTL and HTL epitopes respectively.

### Construction of multi‑epitope vaccine

**Fig. 1:**
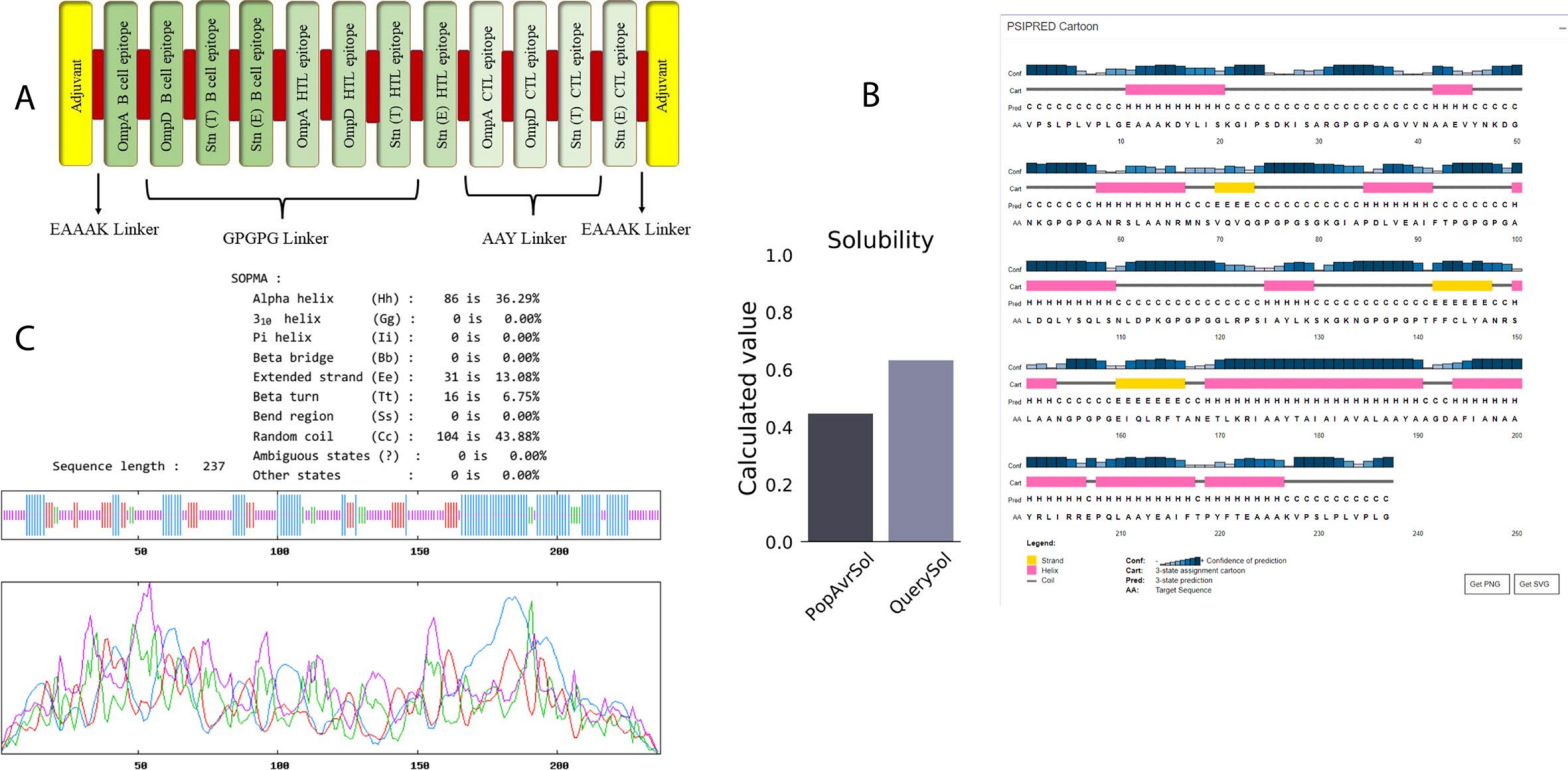
**A.** The final schematic multi-epitope vaccine with 237 amino acids. The adjuvant (Griselimycin) sequence was linked by EAAAK linker at both N and the C terminal of the vaccine the B cell, HTL and CTL epitopes were connected by GPGPG and AYY linkers respectively. **B**. The prediction of secondary structure and solubility of vaccine constructs. **C**. The analysis of secondary structure in SOPMA tool shows that the vaccine contains 36.29% alpha-helix, 13.08% extended strands, 6.75% beta turns and 43.88% random coils

Four epitopes were nominated for each HTL, CTL and B cell to construct vaccine. An adjuvant Griselimycin (APD ID: AP02688) was added to both N and C terminals of the vaccine sequence to increase immunogenicity. In addition, three linkers were used to link epitopes: GPGPG and AAY linkers were used to link B-cell, HTL and CTL epitopes respectively, and EAAAK linkers attach adjuvant to the N and C terminal of epitopes Fig. 1A. Based on the result obtained from Blastp, the vaccine construct and the human proteome were not identical.

### Antigenicity and safety of the constructed vaccine

The result of vaccine's antigenicity was predicted to be 0.81, which defines its highly antigenic properties. In addition, the vaccine model on both AllerTOP and ToxinPred servers was non-allergen and non-toxic respectively.

### Physicochemical analysis of vaccine model

The vaccine model was characterized by a molecular weight of 24.07 kDa with a theoretical isoelectric point (pI) score of 9.49. The vaccine model's half-life was estimated at 100 h for mammalian reticulocytes in vitro and more than 20 h and 10 h in yeast and *E. coli* in vivo respectively. Additionally, the instability index value was 27.57 which indicates the vaccine is stable (> 40: instability). The aliphatic index was 87.09 which shows thermostability [17] and Grand average of hydropathicity (GRAVY) was − 0.051 indicating the hydrophilic properties of the vaccine construct. The protein's solubility score was calculated to be 0.633, indicating that it will be highly soluble when expressed (Fig. 1B). According to SOPMA method results, the entire vaccine sequence had 36.29% alpha helices, 13.08% extended strands, 43.88% random coil and 6.75% beta-turn (Fig. 1C).

### Modeling 3D structure, refinement, and validation of vaccine construct

**Fig. 2:**
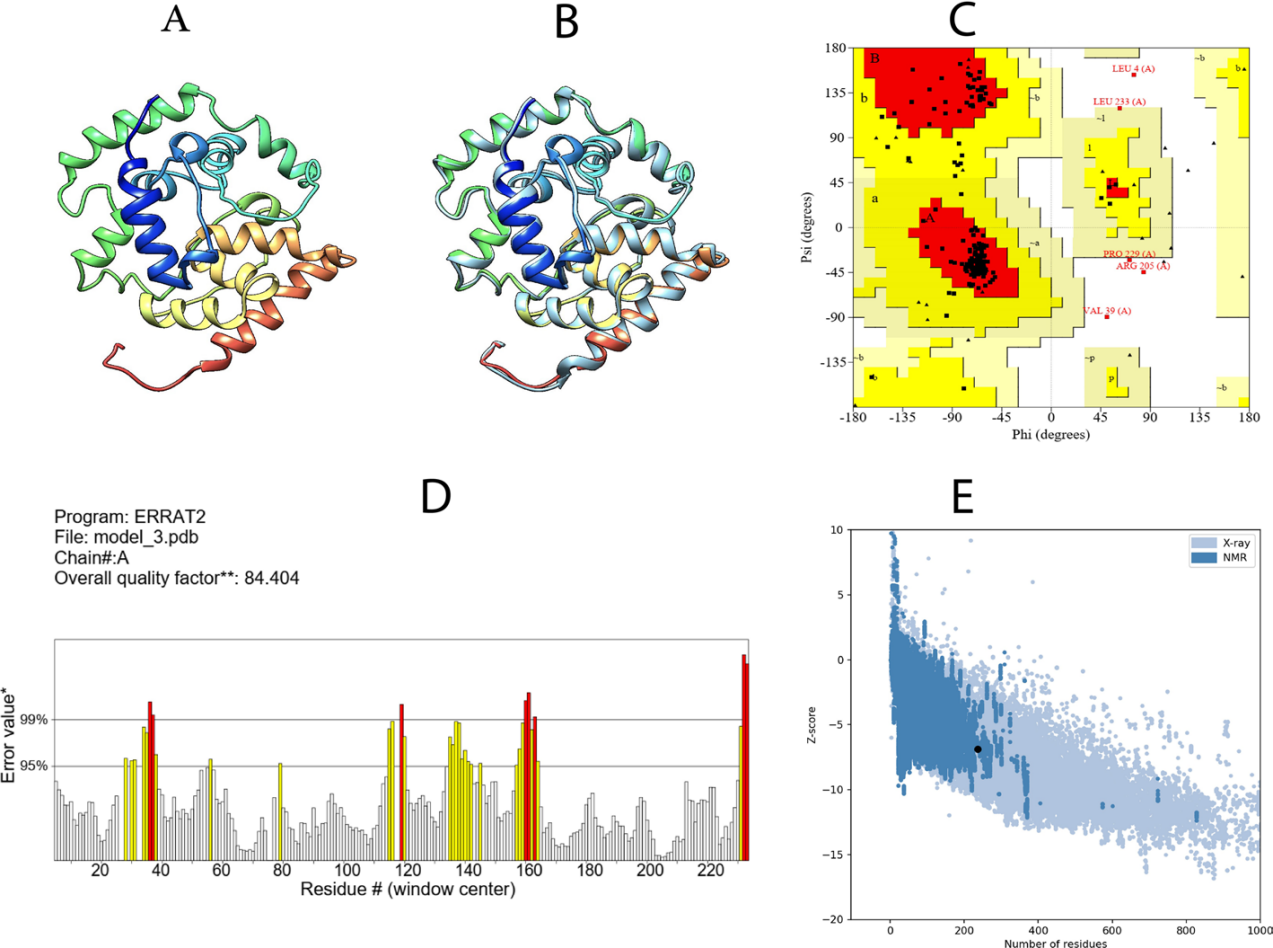
The 3D structure, refinement and validation of multi-epitope vaccine. **A**. The 3D structure of multi-epitope vaccine was predicted by I-TASSER server. **B**. The 3D structure of multi-epitope vaccine after refinement using GalaxyRefine server. **C**. Ramachardan plot indicating 89.3% of the residues were in the most favored region, 8.5% were in the allowed region, and 1.7% were in the disallowed region. **D**. The ERRAT quality factor was 84.4%. **E**. ProSA-web, with a Z score of -6.88

A 3D vaccine structure was built by the I-TASSER server. According to 10 threading templates, 5 tertiary 3D structures were predicted with Z-scores (0.67–1.01) and confidence score (C-score: − 5 to − 4). The C-score is typically in the range of ( − 5, 2), where high score indicates high confidence in the predicted model. Based on these criteria, the best model (C-score: -4) was selected for further analysis. Additionally, this model had TM score and root-mean-square deviation (RMSD) score of 0.19 ± 0.05 and 19.2 ± 2.0 Å respectively (Fig. 2A). The TM-value has been suggested for evaluating the structural similarity of the structures.

The consistency of the vaccine model was improved by using GalaxyRefne web-server. Five models were produced from the refinement of the initial “crude” vaccine model. Model 2 was the most significant model based on structure qualities i.e., GDT-HA (0.9325), RMSD (0.461), and MolProbity (2.117), clash value (11.1), Poor rotamers (0.6) and Rama value (89.8). This model was chosen for further investigation (Fig. 2B).

The refined tertiary structure of the vaccine construct was validated using RAMPAGE web server and Ramachandran plot analysis. In the results, 89.3% of the residues were in the most favored region, 8.5% were in the allowed region, and 1.7% were in the disallowed region (Fig. 2C). In addition, the refinement model had an overall quality factor 84.4% with ERRAT (Fig. 2D). The ProSA-web evaluated the Z score of the vaccine's model to be − 6.88 (Fig. 2E). Overall findings from RAMPAGE, ERRAT, and ProSA-web online tools have validated the quality of the tertiary structure of the vaccine.

### Prediction of discontinuous B cell epitopes

**Fig. 3:**
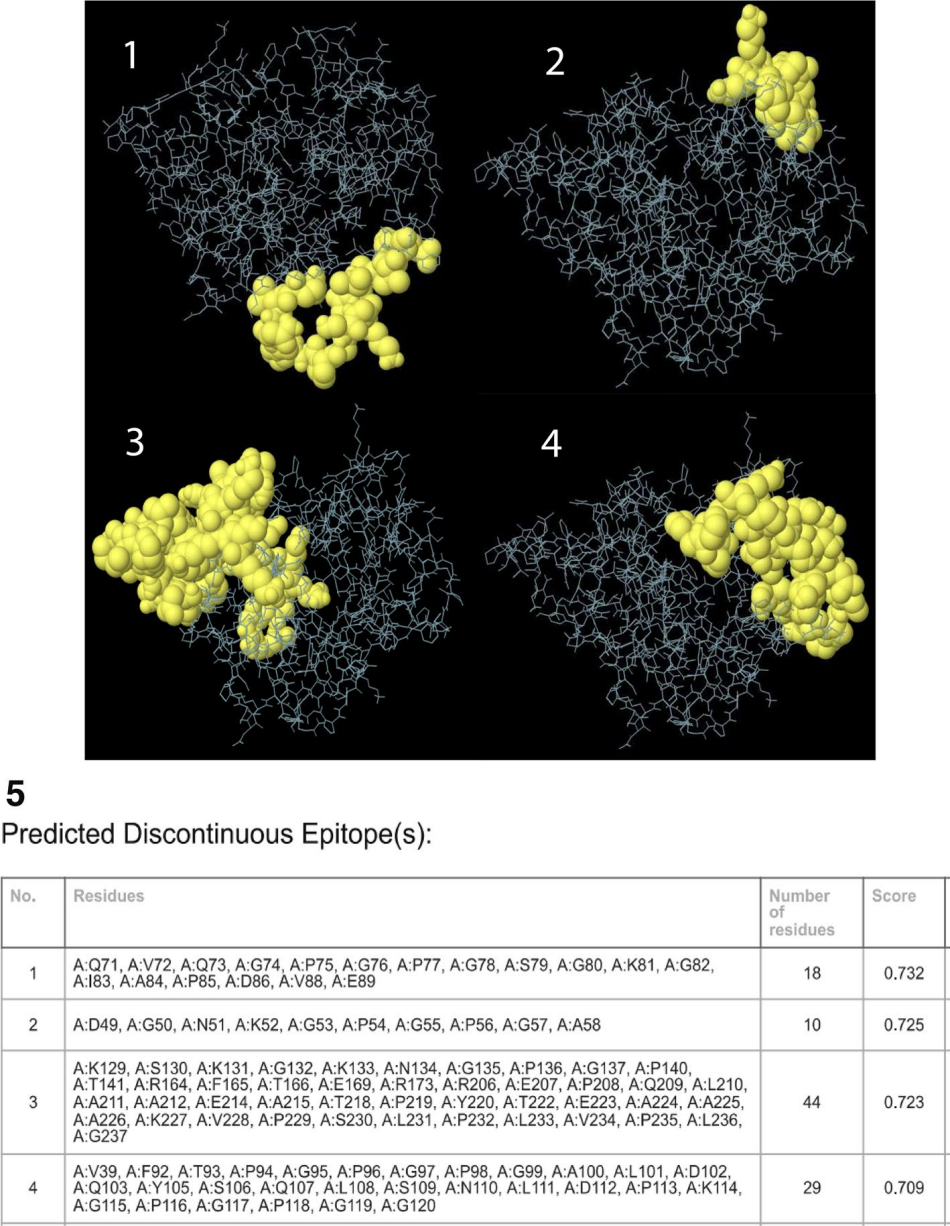
**1–5:** Discontinuous B cell epitopes of multi-epitope vaccine predicted by ElliPro server. **1–4**. The Yellow surface shows discontinuous B cell epitopes. **5**. The residues and score discontinuous B cell epitopes

The results of ElliPro server show that a total of 129 residues were spread over 7 conformational B-cell epitopes, with scores ranging from 0.544 to 0.732. The epitope sizes ranged from 4 to 44. The score value ≥ 0.709 was chosen for discontinuous peptides and they are shown in Fig. 3 (1–5).

### Protein–protein docking

**Fig. 4:**
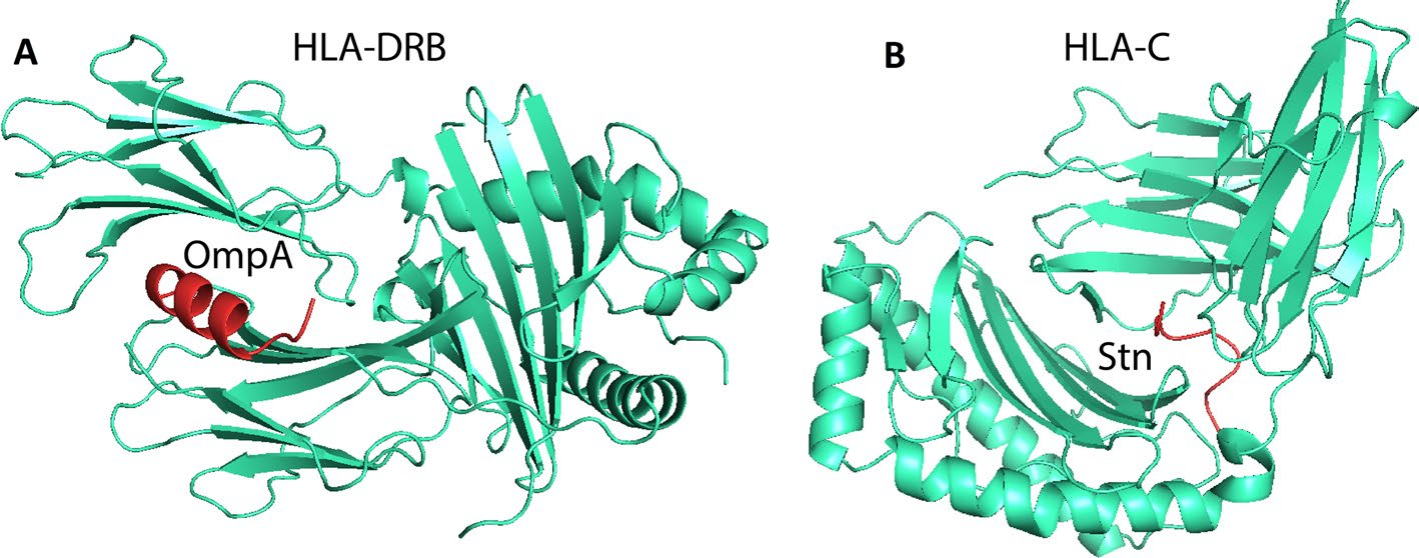
The peptide-protein docking of vaccine model with HLA receptors. **A**. OmpA (ALDQLYSQLSNLDPK: red color) docked with HLA-DRB1 (green color). **B**. Stn (EAIFTPYFTE: red color) docked with HLA-C (green color)

**Fig. 5:**
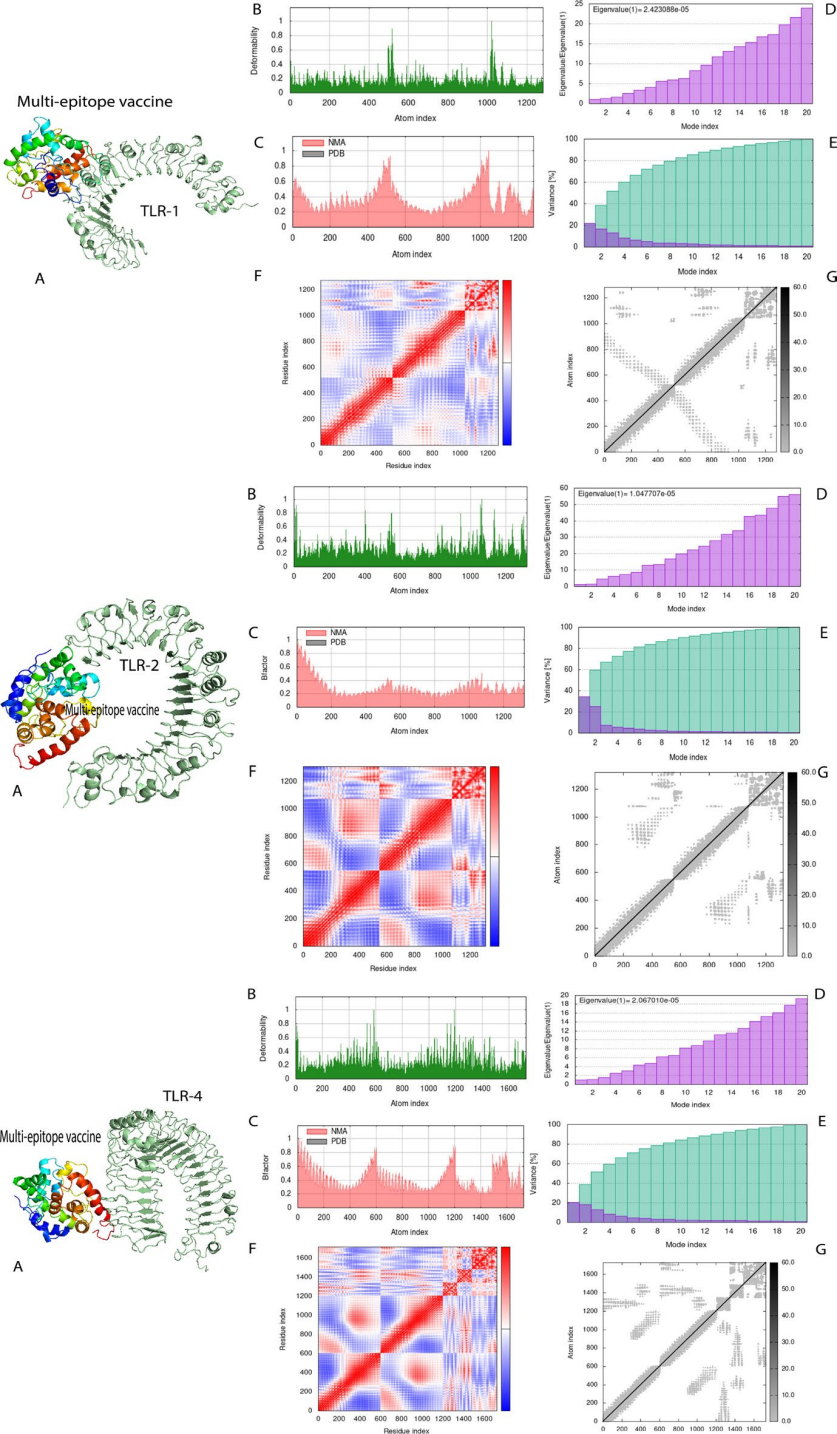
The protein–protein docking of vaccine model with TLR-1, TLR-2, TLR-4 and molecular dynamics simulation. **A**. Protein–protein docking of vaccine model with TLR-1, TLR-2 and TLR-4 by ClusPro 2.0 server **B**. Molecular dynamics simulation: deformability. **C**. B-factor. **D**. Eigenvalues (lower value means easier deformation). **E**. Variance (red color: individual variances and green color: cumulative variances). **F**. Covariance map (red: correlated, white: uncorrelated, blue: anti-correlated). **G**. Elastic network (darker regions mean stiffer regions)

Protein–protein docking between the human immune receptors and the multi-epitope vaccine construct was conducted by ClusPro 2.0 server. The results show that the selected epitopes had a high affinity to bind in a grave of HLA-C with − 693.8 kcal/mol and HLA-DRB1 with − 771.4 kcal/mol (Fig. 4). Since TLR-1, TLR-2 and TLR-4 are highly sensitive to bacterial components, they were selected for docking. The Toll-like receptors detect microbial infection by recognizing microbial components including lipopolysaccharide (TLR-4) and lipoproteins (TLR-1 and TLR-2) [18]. These TLRs are involved in the recognition of *Salmonella* structures and the production of inflammatory cytokines. The best-docked model was chosen from the 30 models on the ClusPro 2.0 server based on its lowest energy value; TLR-1: − 766.6 kcal/mol, TLR-2: − 902.9 kcal/mol and TLR-4: − 863.9 kcal/mol. These findings suggested that the vaccine model could stimulate innate immune receptors and trigger the initiation of further immune responses. The results are presented in Fig. 5.

### Molecular dynamics simulation

The molecular dynamics simulation was performed by iMOD server and normal mode analysis (NMA) to investigate the stability of the vaccine and TLR-1, TLR-2 and TLR-4 complex. The results are shown in Fig. 5. A high degree of deformability was seen in the areas with hinges. In normal mode analysis, the B-factor values are proportional to the root mean square. It was found that the eigenvalue for the TLR-1, TLR-2 and TLR-4 vaccine complex were 2.423088e × 10^–5^, 1.047707 × 10^–5^ and 2.067010 × 10^–5^ respectively. These values indicate the energy required to deform the structure. The lower value means easier deformation. The covariance matrix shows the correlation between pairs of residues. The elastic network model indicates the connection between atoms by springs. All these results suggest that the vaccine model has stable interaction with TLR-1, TLR-2 and TLR-4.

### Immune response simulation

**Fig. 6:**
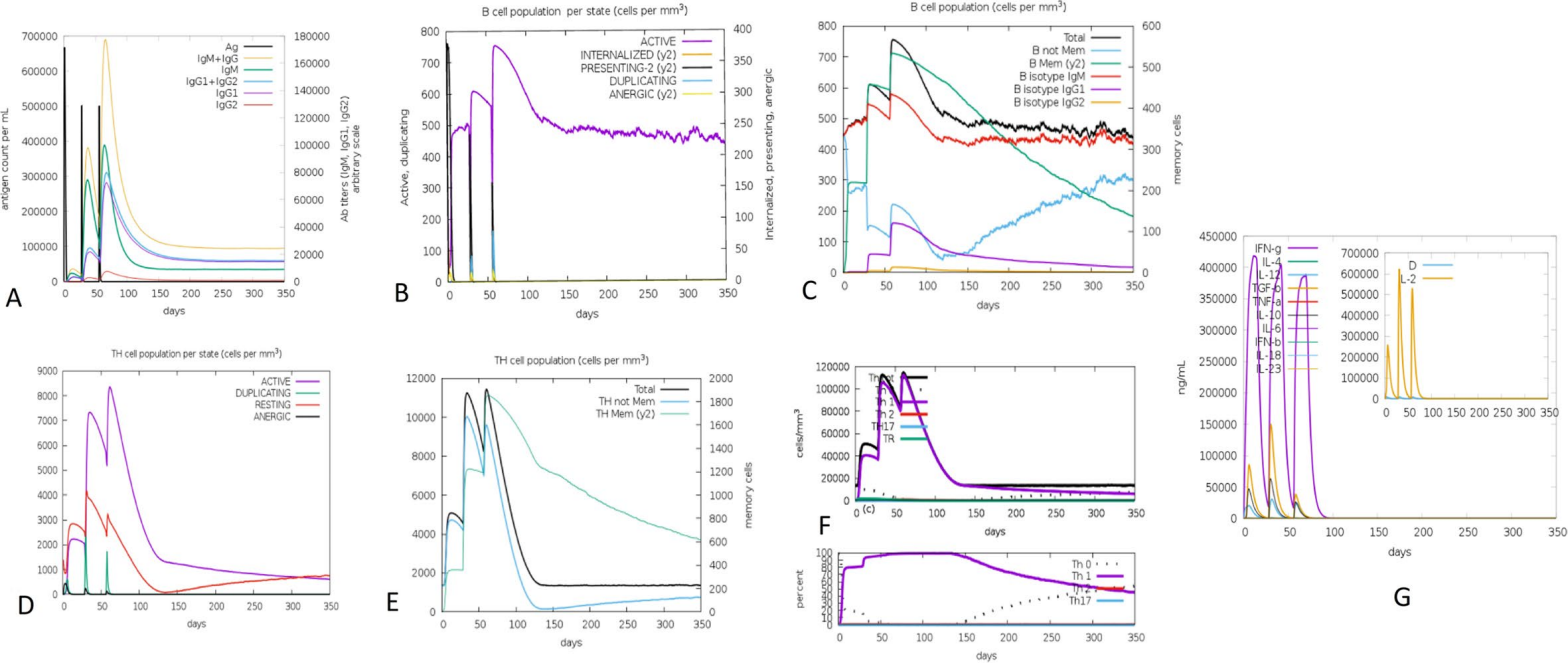
In silico immune system simulations produced by C-ImmSim server. **A**. Increase IgM and IgG responses (cream peak) and decrease antigen (black peak) after the second and third injection **B**. Activation of B cell population (purple peak) **C**. Increase memory B cells (green peak) **D**. Activation of TH cells (purple peak) **E**. Increase memory TH cells (green peak) **(F)**. polarization of T cells response to Th1 (purple peak) **G**. Increase IFN-γ (purple peak) and IL-2 (cream peak) in response to the vaccine

The results of the immune system simulations produced by C-ImmSim server indicated a notable increase in immune responses that were comparable to the real immunological responses. The primary response was determined by an increase in IgM levels. In secondary and tertiary injections, it was seen that B-cell population, IgG1 + IgG2 antibodies, and IgG + IgM antibodies increased, while antigen levels decreased. In addition, the vaccine model could increase the TH population with memory cells and TC cells. IFN-γ and IL-2 production was also elevated after repeated exposure. These results validate the antigenic and immunogenic characteristics of the vaccine model (Fig. 6).

### Vaccine optimization and in‑silico cloning

**Fig. 7:**
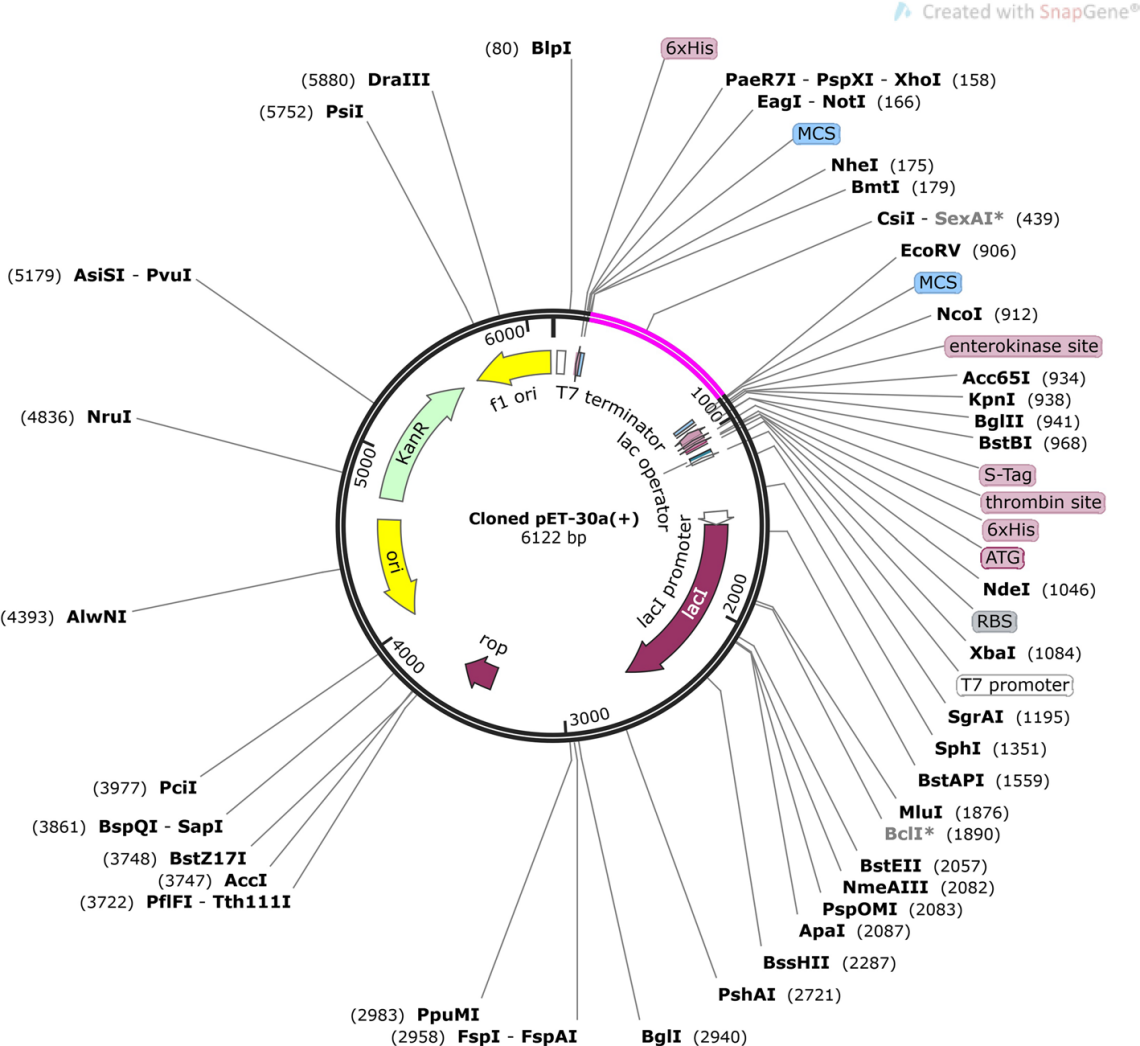
In-silico cloning of the multi-epitope vaccine sequence into the pET30a (+). The magenta area is place the vaccine sequence was inserted

The vaccine amino acid sequence after JCAT optimization had a length of 711 nucleotides. The CAI score was predicted 1.0 and the GC content was 56.6%, which indicates high expression in the *E. coli* K12 [19]. Finally, the optimized vaccine sequence was cloned into plasmid pET30a ( +) and shown in Fig. 7.

## Discussion

*Salmonella* is the most common cause of gastrointestinal disease in healthy people and invasive infections in patients with immune-compromised systems. From the past till now, vaccine development has been considered the most efficient and cost-effective strategy to prevent infectious diseases. For years, scientists have been trying to develop a NTS vaccine, however, there is no licensed vaccine currently available [20]. Since with development of genomics and bioinformatics technologies, the production of chimeric vaccines has been improved [21]. Therefore, due to the advantages of the bioinformatics method including cost-effectiveness, safety and less time duration, researchers have used these techniques for species like viruses, bacteria, and parasites in recent years [22–24].

Recently, Zafar et al. [5] reported the construction of a multi-epitope vaccine candidate against *S*. Typhimurium based on an in-silico analysis. The authors selected B and T-cell epitopes found in TolA (membrane-spanning protein) as the most antigenic protein in *S*. Typhimurium. In another study, Priyadarsini et al. [9] and Ali et al. [16] introduced multi-epitope vaccine candidates against NTS using outer membrane proteins. Since, *Salmonella* Omps are accessible for immune system, essential to cell communication, stable in high temperature and highly immunogenic, they have been considered as potential vaccine candidates in recent years. However, for NTS, no more investigation has yet been conducted. On the other hand, the pathogenicity of *S*. Typhimurium and *S*. Enteritidis were associated to enterotoxin (Stn) [25]. Thus, the present study for the first time suggested a similar workflow for developing a new multi-epitope vaccine candidate based on a combination of outer membrane proteins and *salmonella* enterotoxin. Accordingly, three major proteins OmpA, OmpD and Stn from two serovars *S*. Typhimurium and *S*. Enteritidis were selected for investigation. The NCBI database was used to retrieve protein sequences and BLASTp analysis reveals that OmpA, OmpD share more than 99% identity in *S*. Typhimurium and *S*. Enteritidis. As a result, to construct a multi-epitope vaccine, HTL, CTL and B cell epitopes with high affinity were selected and assembled to create a sequence of the linear protein. OmpC, OmpD and OmpF have previously been used as immunogenic proteins in vaccine model [9]. In this work, OmpA was employed instead of OmpC and OmpF, which has been shown to be highly in vivo immunogenicity. [26]. OmpA is abundant in surface of bacteria and stimulates antibody response along with dendritic cell activation, MHC expression, T cells and IFN-γ production [26, 27]. To improve the efficacy of the vaccine candidate, griselimycin were added as adjuvants at the C and N terminals of the protein. Regarding multi-epitope vaccines, the main features of using linkers are the improved antigen processing and presenting along with modulating rigidity and flexibility of vaccine structure [28, 29]. After binding epitopes, the vaccine model on Vaxijen v2.0 server showed a high score of antigenicity without allergenicity and toxicity.

The final vaccine model contains 237 amino acids with a molecular weight of 24.07 kDa which is optimum for chimeric vaccines [9]. It was suggested that proteins with a molecular weight of less than 110 kDa are better suited since they may be easily extracted and used for vaccine development [30, 31]. The isoelectric point (pI) was 9.49 which shows that the protein is basic, the instability index (II) was 27.57 showing that the protein is stable and has been predicted soluble upon expression [32]. The aliphatic index (87.09) proves that the protein has aliphatic side chains and the Grand average of hydropathicity (GRAVY) was − 0.051 suggesting that the constructed vaccine is hydrophilic [9]. All these parameters indicate that the final vaccine model contains antigenic high-affinity epitopes, along with stability, solubility and thermostability.

In the early stages of *Salmonella* infection due to recognition of bacterial components by TLRs, some hierarchical events initiate: bactericidal activity of macrophages, dendritic cell maturation, and production of inflammatory cytokines and chemokines. There are evidences that TLR-1, TLR2 and TLR4 play an important role in the innate immune response against NTS. Mice deficient in both TLR2 and TLR4 were highly susceptible to *S*. Typhimurium [18, 33].

The Protein–protein docking and molecular dynamics simulations of vaccine model with TLR-1, TLR2 and TLR4 revealed that the vaccine model binds to these TLRs and is probably capable of eliciting an innate and adaptive immune response. Additionally, the outcome of immune stimulation by C-ImmSim server indicates polarization of T cells response to Th1 that helped the humoral response. Previous studies indicate that *Salmonella* infection suppresses humoral immunity on several levels, including B cell lymphopoiesis, MHC-II expression, germinal center development, and the survival of plasma cells [3, 8]. In order to prevent infection, vaccine development against NTS is necessary to generate antibody response before natural infection. Hence, the finding of C-ImmSim server proves the capacity of the vaccine model in triggering antibody response as well as production of T and B cell memory for several months. On the other hand, the level of IFN-γ and IL-2 rose following repeated exposure, which has a positive correlation with the population of TH cells [34]. Research indicates that TH cells are not the only producer cells of IFN-γ, neutrophil and NK cells also contribute to IFN-γ secretion during *S*. Typhimurium infection [3]. As a result, there was a positive association between IFN-γ level and macrophage activation and antigen (vaccine) clearance.

Effective cloning and expression in a suitable host is a key step in the development of a multi-epitope vaccine. The *E. coli* expression systems are the favored host for the expression of recombinant proteins. To achieve high-level expression of the recombinant vaccine in the *E. coli* system, codon optimization was carried out. Since the CAI score was 1.0 and the GC content was between 30 and 60%, it is predicted that the vaccine sequence will be highly expressed in *E. coli* K12.

## Conclusion

This study collectively introduced a multi-epitope vaccine that was developed using the antigenic epitopes of the OmpA, OmpD, and enterotoxin from *S*. Typhimurium and *S*. Enteritidis. The vaccine's antigenicity and safety were confirmed by evaluation of its toxicity, allergenicity, and solubility. Additionally, the interaction of the vaccine with TLRs shows the ability of innate immune system stimulation as well as cellular and humoral responses. This study is the first step in the vaccine production procedure and future in vivo studies will be necessary.

## Methods

### Omps and Stn sequence retrieval

To retrieve Omps and Stn sequence of *S*. Typhimurium and *S*. Enteritidis, NCBI (National Center for Biotechnology Information) database was chosen. Omps and Stn that have been chosen for this research include; *S*. Typhimurium OmpA (Accession No.: AIH09359), *S*. Typhimurium OmpD (Accession No.: QPE03117), *S*. Typhimurium Stn (Accession No.: QCQ29160), *S*. Enteritidis OmpA (Accession No.: UDX53918), *S*. Enteritidis OmpD (Accession No.: UDX71362) and *S*. Enteritidis Stn (Accession No.: QCQ29159). To determine the similarity between proteins in different strains of *S*. Typhimurium and *S*. Enteritidis, Omps and Stn sequences were blasted (BLAST: https://blast.ncbi.nlm.nih.gov).

### Prediction of T lymphocytes epitope

The role of Cytotoxic T lymphocytes (CTL) and helper T lymphocytes (HTL) is very important in the immunogenicity of subunit vaccines particularly in dealing with *Salmonella* infection. To predict CTL and HTL activating epitopes, IEDB (http://tools.iedb.org/main/tcell) server was used to predict peptides that bind to MHC-I and MHC-II, respectively. MHC-I epitopes were predicted based on SMM method with the most frequent alleles in the human population (52 alleles) [35]. For MHC-II epitopes, the 17 most common alleles were selected based on SMM-align prediction method [36]. The length of amino acids was set for MHC-I and MHC-II, 9 and 15 amino acids respectively, and finally, the epitopes with high affinities (IC_50_ < 500 nM) were chosen for further analysis [37–39].

### Prediction of linear B-cell epitopes

B-lymphocyte epitopes are essential for the humoral immune system and antibody production. Therefore, the ABCpred server (https://webs.iiitd.edu.in/raghava/abcpred/ABC_submission.html) and neural network algorithm with a length of 16 amino acids and 0.51 threshold were used to predict linear B cell epitopes of OmpA, OmpD and Stn proteins [40].

### Prediction of immunogenicity, antigenicity, allergenicity and toxicity

The IEDB server (http://tools.iedb.org/immunogenicity) was used to determine the immunogenicity of the MHC-I epitopes [41]. This website defines immunogenicity as peptide-MHC complexes that are recognized by T cells. The VaxiJen v2.0 server (http://www.ddg-pharmfac.net/vaxijen/VaxiJen/VaxiJen.html) was used to assess the antigenicity of multi-epitope vaccine sequence. The server threshold was set as 0.4 for bacteria. This server utilizes the ACC (auto and cross-covariance) to produce an output [42, 43]. To assess whether determined epitopes cause an allergic reaction or not, the AllerTOP v2.0 server (http://www.ddg-pharmfac.net/AllerTOP) was utilized [16, 44]. The AllerTOP uses the ACC transformation of the protein sequence to obtain the closest k value and compare the E descriptors of amino acids [45]. Moreover, the ToxinPred server (https://webs.iiitd.edu.in/raghava/toxinpred/multi_submit.php) was used to check the toxicity of all epitopes [39]. Following the construction of the vaccine similar analyses were again performed.

### Population coverage analysis

The MHC restriction and population coverage of anticipated epitopes for human in different regions were analyzed using the IEDB population coverage tool (http://tools.iedb.org/population/) based on default consensus methods (ANN, SMM, and Combinatorial Library) [43]. This server provides an overview of epitope distribution by calculating the total percentage of people living in a specific region who are possible responses to the chosen epitopes [46].

### Construction of multi‑epitope vaccine sequence

Selected B cell and HTL epitopes were linked by GPGPG linker and CTL epitopes by AAY linker. In addition, a Griselimycin (APD ID: AP02688) was selected as an adjuvant to enhance the immunogenicity of the vaccine and bind through the EAAAK linker [34, 47]. The Griselimycin sequence (APD ID: AP02688) was retrieved from the database (https://aps.unmc.edu/database/anti). Additionally, BLASTp was utilized to evaluate the similarity of the vaccine construct with the human proteome and avoid an autoimmune response [48].

### Analysis of physicochemical properties of the vaccine model

The ProtParam online server (https://web.expasy.org/protparam) was used to predict the physicochemical properties of the vaccine structure including; theoretical isoelectric point (pI), half-life, molecular weight (MW), instability, aliphatic index and grand average of hydropathicity (GRAVY) (14). The Protein Sol database (https://protein-sol.manchester.ac.uk) was also used to determine the solubility of the vaccine construct. The predicted solubility is indicated by the scaled solubility value (QuerySol). Since the population average for the experimental dataset (PopAvrSol) is 0.45, any number greater than 0.45 is anticipated to have a higher solubility than the average soluble *E. coli* protein [40, 49].

### Secondary structure prediction

The secondary structure of the vaccine construct was generated using PSIPRED Property online tool. PSIPRED (http://bioinf.cs.ucl.ac.uk/psipred) is a simple online secondary structure prediction tool that incorporates two feed-forward neural networks which predict transmembrane topology, transmembrane helix, folds, and domains efficiently [50]. The SOPMA method (https://npsa-prabi.ibcp.fr/cgi-bin/npsa_automat.pl?page=/NPSA/npsa_sopma.html) is an online tool used to indicate secondary structure–property. This method correctly predicts 69.5% of secondary structure–property (alpha-helix, beta-sheet, and coil) in proteins database. The combination of SOPMA and a neural networks method (PHD) predicts 82.2% of residues for 74% of co-predicted amino acids [51].

### Tertiary structure prediction

Iterative Threading Assembly Refinement (I-TASSER) server (https://zhanglab.ccmb.med.Umich.Edu/I-TASSER) was used to create a three-dimensional (3D) structure of the vaccine construct. Based on the sequence-to-structure-to-function paradigm, I-TASSER generates three-dimensional (3D) atomic models from multiple threading alignments and iterative structural assembly simulations. The accuracy of the 3D model is provided based on the confidence score (C-score). The C-score is generally in the range between − 5 and 2, wherein a higher number indicates better quality [29].

### Refinement and validation of tertiary structure

To improve the quality of 3D structure, refinement of the vaccine model was carried out with the Galaxy Refine server (https://galaxy.seoklab.org). This server utilizes molecular dynamics simulation to reconstruct the side chains and repack them [52]. To validate the refined tertiary structure of the vaccine model, the PROCHECK server (https://saves.mbi.ucla.edu) was used [53]. The result of this server is the Ramachandran plot, which shows the percentage of residues in favored, allowed and disallowed regions based on dihedral angles psi(ψ) and phi (φ) of each amino acid [54]. Furthermore, ProSA online tool and ERRAT were used for more validation [9, 55].

### Prediction of discontinuous B cell epitopes

Protein folding can put distant residues together and generate discontinuous B-cell epitopes. Since more than 90% of B cell epitopes are discontinuous, the presence of these epitopes in the vaccine structure was predicted by the Ellipro server (http://tools.iedb.org/ellipro/) [56]. The threshold parameters (Minimum residue score 0.5 and Maximum distance 6 Å) were set at default. Based on the 3D structure of the vaccine, this server provides an ellipsoid score to each residue, which is defined as a PI (Protrusion Index) value [43].

### Protein–protein docking and Molecular dynamics simulation

Since the vaccine model must first bind to immunological receptors in order to elicit an immune response. The potential of vaccine docking with immune receptors (MHC and TLR) was evaluated by ClusPro 2.0 server (https://cluspro.bu.edu/login.php) [57]. The PDB files of MHC-I (PDB Id: 5XS3 for HLA-C), MHC-II (PDB Id: 4MDJ for HLA-DRB1), TLR-1 (PDB Id: 6NIH), TLR-2 (PDB ID: 2z7x), and TLR-4 (PDB ID: 3fxi) were download from PDB database (https://www.rcsb.org/). For HLA-C and HLA-DRB1 these receptors docked with epitopes: ‘EAIFTPYFTE’ and ‘ALDQLYSQLSNLDPK’ respectively. To investigate the stability and physical movements of the vaccine-TLR-1, TLR-2 and TLR-4 docked complex, molecular dynamics simulation by iMODS online server (http://imods.Chaconlab.org) was performed [58, 59]. This server uses Normal mode analysis (NMA) in internal (dihedral) coordinates, which can predict the collective motions of proteins. iMODS computes the vaccine-receptor complex's deformability, B-factor, eigenvalues, variance, covariance map, and elastic network [60].

### Immune simulation

The vaccine amino acid sequence was submitted to C-ImmSim server (https://kraken.iac.rm.cnr.it/C-IMMSIM/) in order to determine the immune response profile in the in silico model. C-ImmSim server evaluates humoral and cellular response to vaccine model [61] based on Position-specific scoring matrix (PSSM) and machine learning methods. PSSM stimulates three different anatomical regions of mammals, including Bone marrow, Thymus and tertiary lymphatic organs to define the real-life immune response. To conduct immune stimulation, briefly, all simulation parameters were set as default. Three shots (time steps of injection: 1, 84, and 168) for the four weeks’ interval were administrated with 1050 total simulation steps.

### Vaccine optimization and in‑silico cloning

Finally, the efficacy of the vaccine model in cloning and expression is important for in vitro production. To optimize vaccine construction, first, Java Codon Adaptation Tool (JCat) (http://www.JCat.de/) was performed for best protein expression in *E. coli* strain K12. The levels of protein expression are calculated based on the codon adaptation index (CAI) values (> 0.8) and GC contents (30 to 70%). Then to confirm the expression of vaccine, the optimized vaccine sequence was cloned in *E. coli* plasmid vector pET-30a (+) (within restriction sites of HindIII and BamHI) using the SnapGene software [9].

## Data Availability

The datasets generated and/or analyzed during the current study are available within the manuscript and in NCBI (https://www.ncbi.nlm.nih.gov/) with their accession numbers in this manuscript.

## Abbreviations

NTS: Non-typhoidal *Salmonella*
OMV: Outer membrane vesicles
Omp: Outer membrane protein
GRAVY: Grand average of hydropathicity
JCat: JAVA codon adaptation tool
PSSM: Position-specific scoring matrix
CAI: Codon adaptation index
TLR: Toll-like receptor
pI: Isoelectric point
MW: Molecular weight
